# Administering the maternal appeasing substance to *Bos taurus* and *B. indicus* cattle placed as yearlings in feedlots with different environmental conditions

**DOI:** 10.1093/tas/txaf022

**Published:** 2025-02-11

**Authors:** Reinaldo F Cooke, Shea J Mackey, Izadora S de Souza, Ingo A S Mello, Yasmin M Barreto, Vinicius N Gouvea

**Affiliations:** Department of Animal Science, Texas A&M University, College Station, TX 77843, USA; Department of Animal Science, Texas A&M University, College Station, TX 77843, USA; Department of Animal Science, Texas A&M University, College Station, TX 77843, USA; Ourofino Saúde Animal, Cravinhos, SP 14140-000, Brazil; Texas A&M AgriLife Research and Extension Center, Amarillo, TX 79106, USA; Texas A&M AgriLife Research and Extension Center, Amarillo, TX 79106, USA

**Keywords:** bovine appeasing substance, carcass, finishing cattle, stress, yearlings

## Abstract

Two experiments evaluated growth performance of finishing cattle placed on feed as yearlings, and receiving the maternal bovine appeasing substance (**mBAS**) during stressful management events. In Exp. 1, 240 Angus-influenced yearling steers arrived at the research feedyard on d -1 after a 4-h road transport, and body weight (**BW**) was recorded upon arrival (411.8 ± 2.5 kg). Steers were ranked by arrival BW and received 1 of 2 treatments during initial processing (d 0): 1) 10 mL of a mBAS (Ferappease^®^; FERA Diagnostics and Biologicals; College Station, TX) or 2) 10 mL of mineral oil (**CON**; placebo). Treatments were applied topically to the nuchal skin area (5 mL) and above the muzzle (5 mL). During initial processing, steers were weighed, vaccinated against *Clostridium* spp. and respiratory pathogens, dewormed, implanted, and then distributed into 30 drylot pens according to arrival BW and treatment (n = 15 pens/treatment, 8 steers/pen). Steers were reapplied treatments concurrently with reimplanting on d 75. Initial BW was the average BW on d -1 and 0, final BW was the average of BW recorded on d 138 and 139, and steers were slaughtered on d 139. Steers assigned to mBAS had greater (*P *≤ 0.04) ADG, final BW, and hot carcass weight (**HCW**). Feed intake was greater (*P *≤ 0.05) in mBAS steers, but mostly after reimplanting (treatment × day; *P* < 0.01). Carcass yield grade, backfat thickness, marbling score, and % Choice or better were greater (*P *≤ 0.05) in mBAS steers. In Exp. 2, Nelore (*Bos indicus*) yearling bulls (n = 2,626) arrived at a feedyard (d 0) after a 96-h road transport, and BW was recorded upon arrival (shrunk BW = 352.9 ± 0.6 kg). Bulls were assigned to receive mBAS or CON as in Exp. 1 during initial processing on d 0 (10 pens/treatment; ~130 bulls/pen) which included deworming and vaccination against *Clostridium* spp. and respiratory pathogens. Bulls were on feed for 96.0 ± 1.0 d until slaughter. Bull ADG was calculated using arrival BW considering a 14% shrink and final BW recorded when loading bulls to slaughter. Bulls assigned to mBAS had greater (*P *≤ 0.04) ADG, feed intake, final BW, and HCW. Morbidity and mortality rates due to respiratory disease were less (*P *= 0.02) in mBAS bulls. Collectively, mBAS administration during stressful management events improved growth performance, mainly by increasing feed intake, in finishing cattle placed on feed as yearlings in feedlot systems typical of US (Exp. 1) and tropical regions (Exp. 2).

## Teaser

Feedlot cattle placed on feed as yearlings, despite not being considered high-risk for disease development, are exposed to stressors during the finishing period. Two experiments evaluated the maternal bovine appeasing substance, an analogue of the bovine appeasing pheromone, as a strategy to alleviate such stressors and improve their feedlot performance, health, and carcass quality.

## INTRODUCTION

The maternal bovine appeasing substance (**mBAS**) is a mixture of fatty acids that replicate the composition of the original bovine appeasing pheromone, and shown to alleviate the physiological consequences elicited by stressful management procedures (Cappellozza and Cooke, 2022). Accordingly, mBAS administration improved health and growth performance of feedlot cattle (Colombo et al., 2020; Fonseca et al., 2021; Pickett et al., 2024) given the negative consequences of stress to cattle productivity and immunocompetence (Cooke, 2017). Most of these studies, however, focused on mBAS administration to high-risk calves during feedlot receiving. Therefore, research warranted to determine the effects of mBAS administration to cattle placed on feedlots as yearlings, as these represent of substantial portion of finishing cattle in the US and across the world (Endres and Schwartzkopf-Genswein, 2018; Greenwood, 2021).

Despite not being considered high-risk, yearling cattle can also be exposed to stressors during the finishing period that affect their growth performance and carcass quality (Cooke, 2017; Kumar et al., 2023). Such stressors include commingling, exposure to novel managerial procedures, and handling for initial processing and reimplant (Cooke, 2017; Smith et al., 2020; Sperber et al., 2024). Long road-transport is another source of stress for feedlot cattle, particularly in tropical countries where *Bos indicus* cattle predominate (Grandin, 2019). *Bos indicus* breeds are highly susceptible to stressors associated with transitioning from grazing to feedlot systems compared with *B. taurus*, particularly when *B. indicus* cattle are fed as bulls (Cooke et al., 2020; Mota-Rojas et al., 2024). Therefore, this study evaluated mBAS administration to Angus-influenced yearlings steers exposed to typical US feedyard management (Exp. 1) and to Nelore (*B. indicus*) yearlings bulls transported for 96 h prior to feedlot arrival in a tropical environment (Exp. 2). We hypothesized that mBAS administration will improve health and growth performance of finishing cattle placed on feed as yearlings in both production systems.

## MATERIALS AND METHODS

Experiment 1 was conducted at the Texas A&M AgriLife Research feedlot (Bushland, TX), and cattle were cared for in accordance with acceptable practices and experimental protocols reviewed and approved by the West Texas A&M University Institutional Animal Care and Use Committee (2024.04.00). Experiment 2 was conducted at a commercial feedyard (BLT Confinamento; Guaíra/SP, Brazil), and bulls were cared for in accordance with acceptable practices as outlined in the Guide for the Care and Use of Agricultural Animals in Agricultural Research and Teaching (FASS, 2020).

### Experiment 1

#### Animals and treatments.

A total of 240 Angus-influenced yearling steers were purchased from a commercial auction facility in Beaver, OK, and used in this experiment. Steers were backgrounded on pasture from weaning until the time of purchase, whereas no other detailed information on health and management history was available. On the day of purchase (d -1; 0600 h), steers were loaded into 5 commercial livestock trailers and transported for 280 km (4 h) to the experimental facility (Bushland, TX). Upon arrival, steers were unloaded and their arrival body weight (**BW**) recorded (average ± SEM = 411.8 ± 2.5 kg), and then rested as a single group for 24 h with ad libitum access to wheat hay, water, and a commercial mineral + vitamin mix (Table 1). On d 0, steers were ranked according to arrival BW and assigned to receive mBAS (Ferappease^®^; FERA Diagnostics and Biologicals; College Station, TX; n = 120) or placebo (mineral oil; **CON**, n = 120) in a manner that treatment groups had equivalent arrival BW. The active ingredient of mBAS is based on a proprietary mixture of fatty acids including palmitic, oleic, and linoleic acids, added at 10% of the excipient and estimated to remain in treated animals for 15 d (Schubach et al., 2020). Steers were segregated by treatment (2 groups) and immediately processed again for initial processing and treatment administration, with CON steers processed first to avoid cross-contamination during treatment application (Pickett et al., 2024). Treatments (10 mL) were applied topically to the nuchal skin area (5 mL) and above the muzzle (5 mL) using the applicator provided by the manufacturer (FERA Diagnostics and Biologicals).

**Table 1.** Composition and nutritional profile of the total mixed ration offered for ad libitum consumption to steers during Exp. 1

Item	Step 1	Step 2	Step 3	Finishing
Composition, dry matter basis				
Corn stalks, %	39.4	29.7	19.9	12.2
Steam-flaked corn, %	36.8	46.7	57.9	66.4
Sweet Bran, %	14.3	14.4	14.5	14.5
Dried distillers’ grains, %	5.54	3.85	1.66	0.00
Crude corn oil, %	0.00	1.21	1.83	2.45
Urea	0.482	0.605	0.730	0.855
Mineral-vitamin mix,^1^ %	3.43	3.44	3.46	3.48
Nutritional profile,^2^ dry matter basis				
Net energy for maintenance, Mcal/kg	1.67	1.84	1.87	2.17
Net energy for gain, Mcal/kg	1.06	1.22	1.23	1.48
Neutral detergent fiber, %	37.7	31.5	29.5	22.0
Crude protein, %	13.8	14.0	12.7	13.5
Crude fat, %	3.11	4.07	4.12	4.95

^1^Containing 27.3% calcium carbonate, 22.6% ground corn, 20.6% magnesium sulfate, 17.3% monocalcium phosphate, 7.02% added salt, 4.03% potassium chloride, 0.335% Rumensin 90 (Elanco Animal Health, Greenfield, IN), 0.249% manganese sulfate, 0.143% vitamin E (500 IU/g), 0.138% zinc oxide, 0.080% copper sulfate, 0.042% sodium selenite, 0.005% vitamin A (1,000,000 IU/g), 0.0015% cobalt carbonate, 0.0012% ethylenediamine dihydroiodide, and 0.0011% vitamin D (500,000 IU/g).

^2^Wet chemistry procedures by a commercial laboratory (Dairy One Forage Laboratory, Ithaca, NY). Calculations for net energy for maintenance and gain used equations from the NASEM (2016). Steers were offered the step 1 diet from day 0 to 7, the step 2 diet from day 8 to 14, and the step 3 diet from day 15 to 21, and the finishing diet from day 22 to 138 (slaughter on day 139).

During initial processing, steers received vaccines against *Clostridium chauvoei*, *C. septicum*, *C. novyi* type B, *C. haemolyticum, C. tetani* and *C. perfringens* types C and D (Covexin 8; Merck Animal Health, Madison, NJ), *Mannheimia haemolytica*, bovine respiratory syncytial virus, bovine herpesvirus-1, bovine viral diarrhea virus 1 and 2, and parainfluenza-3 virus (Vista Once SQ; Merck Animal Health), anthelmintic (Safe-Guard, Merck Animal Health), and a growth-promoting implant (Synovex Choice^®^ containing 100 mg trenbolone acetate/14 mg estradiol benzoate; Zoetis, Florham Park, NJ). Steers within treatment were ranked again by arrival BW and allocated to 1 of 30 drylot pens (150 m^2^; 8 steers/pen; n = 15/treatment), in a manner that all pens had equivalent arrival BW. Pens were arranged in 4 rows (2 rows of 6 pens each, and 2 rows of 9 pens each), and rows were alternately assigned to mBAS and CON pens to preserve distance between pens from different treatments (Colombo et al., 2020). From d 0 to 138, steers had free-choice access to water and a total-mixed ration (**TMR;**Table 1) that was offered once daily (0700 h). On d 75, all steers were reimplanted (Synovex Plus^®^ containing 200 mg trenbolone acetate/28 mg estradiol benzoate; Zoetis) and received another 10-mL dose of mBAS or CON according to treatment (5 mL in nuchal skin area and 5 mL above the muzzle). The CON pens were processed first followed by the mBAS pens to avoid cross-contamination. On d 139 (1000 h), all steers were loaded into 8 commercial livestock trailers (2 pens/treatment in each trailer, but for 1 trailer that had 1 pen/treatment) and transported to a packing facility (Tyson Foods; Amarillo, TX) for slaughter on the same day.

#### Sampling.

Samples of the TMR were collected weekly, pooled across weeks, and analyzed for nutrient content by a commercial laboratory (Cumberland Valley Analytical Services; Waynesboro, PA) for DM at 105 °C (method 930.15; AOAC, 2006), total nitrogen (N; Leco FP-528 Nitrogen Combustion Analyzer; Leco Corp., St. Joseph, MI), neutral detergent fiber using sodium sulfite and heat stable amylase (Van Soest et al., 1991), acid detergent fiber and lignin (method 973.18; AOAC, 2006), ash (method 942.05; AOAC, 2006), and crude fat (method 2003.05; AOAC, 2006). Steers were also weighed during initial processing (unshrunk BW; day 0), and BW from day −1 and 0 were averaged as initial BW. Final BW was calculated by averaging unshrunk BW collected on day 138 and at the time of loading on day 139. Steer average daily gain (**ADG**) was calculated using initial and final BW. Initial and final BW were calculated using unshrunk BW collected over 2 consecutive days to minimize BW variability due to gut fill (Watson et el., 2013).

Feed bunks were monitored at 0630, 1300, and 1800 h daily to make assessments for the next feed calls (Pritchard and Bruns, 2003). The amount of feed offered to each pen was adjusted based on the TMR intake of the previous day, and bunks were managed for a maximum of 5% orts. Orts were removed weekly, weighed, sampled for dry matter (**DM**) determination (dried for 96 h at 50 °C in forced-air ovens). Feed intake (DM basis) was evaluated by recording daily TMR offer and weekly orts, divided by the number of steers within each pen, and expressed as kg per steer/d. Gain to feed (**G:F**) ratio was calculated using total BW gain and total feed intake of each pen during the experimental period. Steers were observed daily for health conditions, including signs of bovine respiratory disease (**BRD**) and digestive disorders as in Colombo et al. (2021). Hot carcass weight (**HCW**) was recorded upon slaughter on day 139, whereas trained personnel assessed carcass characteristics including backfat thickness at the 12^th^-rib, marbling, and *Longissimus muscle* (**LM**) area after a 24-h chill.

### Experiment 2

#### Animals and treatments.

A total of 2,626 Nelore (*B. indicus*) yearling bulls were purchased from a commercial ranch near Redenção/PA (Brazil) and used in this experiment. As in Exp. 1, bulls were backgrounded pasture from weaning until the time of purchase, but no other detailed information on health and management history was available. Bulls were loaded into commercial livestock trailers and transported for 1,800 km (96 h) to the commercial feedyard (Guaíra/SP, Brazil). More specifically, all bulls were loaded and transported within a 14-d period with approximately 260 bulls loaded into 4 trailers/day. At the time of loading (day −4; 0800 h), bulls received a metaphylaxis treatment (15 mg/kg of BW of tilmicosin; Micotil, Elanco Saúde Animal; São Paulo, Brazil). Upon arrival (day 0), bulls were unloaded, their arrival BW recorded (average shrunk BW ± SEM = 352.9 ± 0.6 kg) and then immediately assigned to initial processing and treatment administration. The first ~130 bulls to be unloaded (2 trailers) were processed, assigned to CON as in Exp.1 and allocated to a drylot pen (3,600 m^2^). The remaining ~130 bulls (2 trailers) were processed, assigned to mBAS as in Exp.1 and allocated to another drylot pen (3,600 m^2^). The next unloading day, the first ~130 bulls unloaded were assigned to mBAS and the remaining bulls to CON. This process was repeated over the 14-d period, resulting in 10 pens assigned to CON and 10 pens assigned to mBAS (~130 bulls/pens).

During initial processing, bulls were vaccinated against *C. chauvoei*, *C. septicum*, *C. novyi*, *C. sordelli, C. perfringens* types B and D, and *C. botulinum* types C and D (Resguard Multi; Vaxxinova, São Paulo/SP, Brazil), *M. haemolytica*, *Haemophilus somnus*, *Pasteurella multocida*, bovine herpesvirus-1, bovine viral diarrhea virus 1 and 2, and parainfluenza-3 virus (Providean Respiratória^®^; Agener União Saúde Animal, São Paulo/SP, Brazil), and also received an anthelmintic (Frigoboi^®^ Facilite with 0.5% Abamectin; JA Saúde Animal, Patrocínio Paulista/SP, Brazil). Pens were arranged in 4 rows of 5 pens each, and rows alternately assigned to mBAS and CON pens to preserve distance between pens from different treatments (Colombo et al., 2020). Bulls had free-choice access to water and a TMR (Table 2) offered twice daily (0800 and 1500 h) for (average ± SEM) 96.0 ± 1.0 d, given that pens were slaughtered according to the management plan of the feedyard. On the day of slaughter, bulls from the same pen were loaded into 3 trailers and transported to a packing facility (Marfrig S/A; Promissão/SP, Brazil).

**Table 2.** Composition and nutritional profile of the total mixed ration offered for ad libitum consumption to bulls during Exp. 2

Item	Step 1	Finishing
Composition, dry matter basis		
Corn silage, %	52.7	34.4
Dried distillers’ grains, %	25.0	24.0
Ground corn, %	13.0	21.1
Cottonseed hulls, %	7.00	14.9
Tomato residue, %	0.00	2.00
Mineral-vitamin mix,^1^ %	1.80	1.80
Culled grain corn, %	0.00	1.48
Urea	0.500	0.320
Nutritional profile,^2^ dry matter basis		
Net energy for maintenance, Mcal/kg	1.78	1.90
Net energy for gain, Mcal/kg	1.17	1.25
Neutral detergent fiber, %	46.4	41.7
Crude protein, %	13.9	14.2
Crude fat, %	8.42	8.50

^1^Containing 16.5% calcium, 2.30% phosphorus, 4.0% sodium, 1.50% magnesium, 1.40% sulfur, 25 ppm of cobalt, 420 ppm copper, 25 ppm iodine, 810 ppm manganese, 15 ppm selenium, 1,500 ppm zinc, 72,000 IU/kg vitamin A, 14,370 IU/kg vitamin D3, 500 IU/kg vitamin E, 714 ppm of monensin (Rumensin^TM^; Elanco Saúde Animal; São Paulo, Brazil), and 714 ppm of virginiamycin (V-MAX^®^; Phibro Saúde Animal; Bragança Paulista/SP, Brazil).

^2^Wet chemistry procedures of ingredients by a commercial laboratory (TecnoBeef Indústria e Comércio S. A.; Altair/SP, Brazil). Calculations for net energy for maintenance and gain used equations from the NASEM (2016). Bulls were offered the step 1 diet from day 0 to 20, and the finishing diet from day 21 to slaughter (average ± SEM = 96.0 ± 1.0 d on feed).

#### Sampling.

Samples of all TMR ingredients were collected prior to the beginning of the experiment, and analyzed for nutrient content by a commercial laboratory (TecnoBeef Indústria e Comércio S. A.; Altair/SP, Brazil) using the same methodologies as in Exp. 1. Bull BW was recorded individually at the time of initial processing (shrunk BW, day 0) and a 14% transport shrink was considered (Schwartzkopf-Genswein et al., 2016); therefore, initial BW was calculated as shrunk BW (day 0) × 1.14. Final BW was recorded individually when loading for slaughter (unshrunk). Bull ADG was calculated using initial and final BW.

Feed bunks were monitored at 0700 h daily to make assessments for the next feed calls (Pritchard and Bruns, 2003) as in Exp. 1, and bunks managed for a maximum of 5% orts. Orts were removed weekly, weighed, sampled for DM determination (dried for 48 h at 105 °C in forced-air ovens). Feed intake (DM basis) was evaluated by recording daily TMR offer and weekly orts, divided by the number of bulls within each pen, and expressed as kg per bull/d. The G:F ratio was calculated using total BW gain and total feed intake of each pen during the experiment. Bulls were observed daily for health conditions as in Exp. 1, and treated for BRD with florfenicol antibiotic + flunixin meglumine (Resflor Gold, Merck Animal Health) at 1 mL/7.6 kg of BW. Hot carcass weight was recorded upon slaughter, whereas no other carcass trait was assessed.

### Statistical Analyses

Data from both experiments were analyzed using a complete randomized design using pen as the experimental unit, and Satterthwaite approximation to determine the denominator degrees of freedom for tests of fixed effects. Quantitative data were analyzed using the MIXED procedure of SAS (SAS Inst. Inc., Cary, NC), whereas binary data were analyzed using the GLIMMIX procedure of SAS (SAS Inst. Inc.) with a binomial distribution and logit link function. All models included pen(treatment) and animal(pen) as random variables, but for pen-based assessments (feed intake and G:F) that used pen(treatment) as random variable. Model statements contained the fixed effect of treatment, in addition to day and the treatment × day interaction for feed intake. The specified term for the repeated statement was day, with pen(treatment) as subject, and the covariance structure used was first-order autoregressive according to the smallest Akaike information criterion. In Exp. 2, initial BW and DOF were included as independent covariates in all analyses because experimental units and treatments were not balanced for these variables on day 0. Results from Exp.1 are reported as least square means, or covariately-adjusted least square means for Exp. 2. Significance was set at *P* ≤ 0.05 and tendencies were determined if *P* > 0.05 and ≤ 0.10.

## RESULTS AND DISCUSSION

### Experiment 1

**Figure 1.**
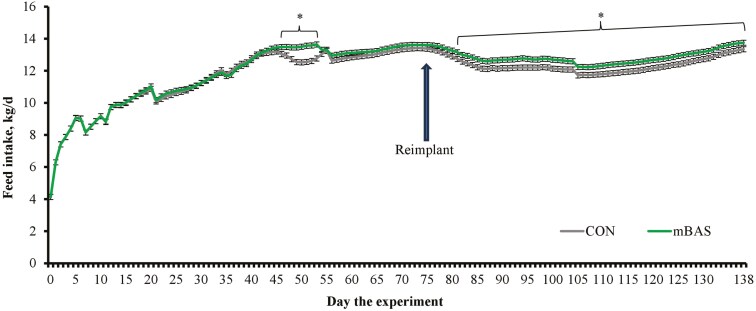
Feed intake of yearling steers (Exp. 1) administered the maternal bovine appeasing substance (**mBAS**; n = 15) or mineral oil (**CON**; n* *= 15) during a 138-d finishing period. Prior to initial processing on day 0 that included a growth-promoting implant (Synovex Choice^®^; Zoetis, Florham Park, NJ), steers individually received 10-mL of the mBAS (FerAppease^®^; FERA Diagnostics and Biologicals; College Station, TX) or mineral oil (CON). On day 75, all steers were reimplanted (Synovex Plus^®^; Zoetis) and received another 10-mL dose of mBAS or CON according to treatment. Treatments were applied topically to the nuchal skin area (5 mL) and above the muzzle (5 mL) of each steer. Feed intake was evaluated by recording daily offer of the total mixed ration and measuring orts weekly from each pen, which was divided by the number of steers within each pen and expressed as kg per steer/d on dry matter basis. A treatment × day interaction was detected (*P* < 0.01). Within days, * *P* ≤ 0.05.

Initial BW did not differ between CON and mBAS steers as designed (*P *= 0.84). Steer administered mBAS had greater (*P *≤ 0.04) ADG and final BW compared with CON steers (Table 3). A treatment × day interaction was detected (*P* < 0.01) for feed intake (Figure 1), which was greater (*P* < 0.05) for mBAS steers from day 47 to 54 and from day 82 until slaughter. Mean feed intake during the experiment was also greater (*P *= 0.04) for mBAS vs. CON steers, and G:F did not differ (*P *= 0.49) between treatments (Table 3). Hence, the greater ADG and final BW of mBAS steers resulted from their increased feed intake, particularly after reimplanting on day 75. No incidence of BRD or morbidity events that required intervention were noted, although mortality was observed but did not differ (*P *= 0.46) between treatments (3.33% for CON and 1.67% for mBAS steers; SEM = 1.57). The lack of morbidity events including BRD incidence was expected as steers used in this experiment were not considered high-risk given their age, short transportation distance, and BW at arrival (Duff and Galyean, 2007; Cooke, 2017). All mortalities occurred overnight and were attributed to severe digestive disorders (Colombo et al., 2021) or congestive heart failure (Johnson et al., 2021).

**Table 3.** Performance parameters during a 138-d finishing period of yearling steers (Exp. 1) administered the maternal bovine appeasing substance (**mBAS**; n = 15) or mineral oil as placebo (**CON**; n* *= 15) ^1^

Item	CON	mBAS	SEM	*P-value*
Initial BW,^2^ kg	412.1	411.2	3.6	0.84
Final BW,^2^ kg	722.6	733.8	3.9	0.04
Average daily gain, kg/d	2.25	2.34	0.03	0.03
Feed intake,^3^ kg/d	11.8	12.2	0.1	0.04
Gain to feed,^4^ kg/kg	0.189	0.190	0.001	0.49

^1^Steers were purchased (day −1) from a commercial auction facility, transported for 280 km (4 h) and unloaded at the research yard. Steers were maintained as a single group for 24 h with access to fresh water and wheat hay. During processing on day 0, steers individually received 10-mL of the mBAS (Ferappease^®^; FERA Diagnostics and Biologicals; College Station, TX) or mineral oil (CON). Treatments were applied topically to the nuchal skin area (5 mL) and above the muzzle (5 mL) of each steer. Initial processing included administration of vaccines, anthelmintic, and growth-promoting implant (Synovex Choice^®^; Zoetis, Florham Park, NJ). On day 75, all steers were reimplanted (Synovex Plus^®^; Zoetis) and received another 10-mL dose of mBAS or CON according to treatment (5 mL in nuchal skin area and 5 mL above the muzzle).

^2^Body weight (**BW**) recorded on day −1 and 0 (unshrunk) were averaged as initial BW. Final BW was calculated by averaging unshrunk BW collected on day 138 and 139.

^3^Feed intake was evaluated by recording daily offer of the total mixed ration and measuring orts weekly from each pen, which was divided by the number of steers within each pen and expressed as kg per steer/d.

^4^Gain to feed was calculated using total BW gain (based on initial and final BW), and total feed intake (kg of dry matter) of each pen during the 138-d finishing period.

The stress elicited by processing finishing cattle for reimplant decreases feed intake and subsequent growth performance (Smith et al., 2020; Sperber et al., 2024). Results from Exp. 1 support this rationale as feed intake immediately decreased after day 75 for both treatment groups (day effect; *P *< 0.01). Administering mBAS maintained feed intake of mBAS steers greater compared with CON from day 82 until slaughter. If feed intake was improved due to mBAS application on day 75, or also due to residual effects from mBAS application on day 0 deserves further investigation. Nonetheless, the stress resultant from feedlot entry and subsequent handling events are known to impair feed intake (Cooke, 2017), whereas mBAS mitigates the physiological consequences of stress that depress appetite (Cappellozza and Cooke, 2022). In previous studies with high-risk and newly weaned cattle, mBAS administration improved ADG mainly by increasing feed efficiency (Colombo et al., 2020; Cooke et al., 2020; Schubach et al., 2020). Intake is already limited in high-risk calves (Duff and Galyean, 2007), which may reduce the benefits of mBAS to this trait. This experiment, however, is the first to evaluate mBAS administration to steers placed on feedyards as yearlings and exposed to the stress of reimplant. The physiological mechanisms affected by mBAS administration were previously described (Cappellozza and Cooke, 2022), particularly stress-induced neuroendocrine and inflammatory responses that modulate feed intake and growth (Cooke, 2017). Hair cortisol concentrations and acute-phase proteins are physiological variables affected by stress, but that require frequent sampling to characterize the mBAS effects (Schubach et al., 2020; Picket et al., 2024). These variables were not evaluated herein to minimize cattle handling, and ensure that this experimental design was representative of commercial feedyards systems.

Hot carcass weight was greater (*P *= 0.04) in mBAS compared with CON steers (Table 4) corroborating treatment differences in ADG and final BW. Carcass dressing did not differ (*P *= 0.90) between treatments (Table 4). Mackey et al. (2024) reported that carcass dressing can be increased by mBAS when administration occurred one week prior to slaughter; however, this approach was not investigated herein. Yield grade and backfat thickness were greater (*P *≤ 0.04) in mBAS steers (Table 4), which can be directly associated with their greater HCW (Owens et al., 1993; Owens and Gardner, 2000; Mayer et al., 2024). In turn, LM area did not differ (*P *= 0.87) between treatments (Table 4). Yield grade is positively associated with HCW and backfat thickness, but negatively associated with LM area (Mayer et al., 2024). Hence, treatment differences in yield grade were mostly driven by greater HCW and backfat thickness of mBAS steers. Carcass marbling was improved (*P *= 0.01) in mBAS compared with CON steers, resulting in a greater (*P *= 0.05) proportion of mBAS steers that graded Choice or Prime (Table 4). The proportion of carcasses graded as Premium Choice was increased (*P *= 0.03), whereas the proportion of carcasses graded as Select was decreased (*P *= 0.05) in mBAS steers (Table 4). Improved marbling of mBAS steers can also be associated with their greater ADG and subsequent HCW at slaughter, as well as enhanced lipogenesis due to feed intake (Nguyen et al., 2021). Intramuscular fat deposition in cattle can be heightened according to intake of insulinogenic diets, such as the TMR utilized in this experiment (Pethick et al., 2004). Moreover, stress-induced neuroendocrine and inflammatory responses, such as cortisol and acute-phase reactions, can impair marbling by promoting lipolysis while limiting lipogenesis (Park et al., 2018). These physiological responses were not evaluated as previously mentioned, but were alleviated by mBAS administration in previous studies (Cappellozza and Cooke, 2022; Mackey et al., 2024; Pickett et al., 2024).

**Table 4.** Carcass parameters of yearling steers (Exp. 1) administered the maternal bovine appeasing substance (**mBAS**; n = 15) or mineral oil as placebo (**CON**; n* *= 15) during a 138-d finishing period^1^

Item	CON	mBAS	SEM	*P-value*
Hot carcass weight, kg	440.3	447.3	2.5	0.04
Dressing, %	60.9	60.9	0.1	0.90
Backfat, cm	1.38	1.51	0.04	0.02
*Longissimus muscle* area, cm^2^	106.8	106.6	0.9	0.87
Marbling score	481.7	508.1	7.3	0.01
Yield grade	2.78	2.97	0.07	0.04
Carcasses graded Choice or Prime, %	87.9	94.9	2.6	0.05
Carcasses graded Select	12.1	5.08	2.58	0.05
Carcasses graded Choice	51.7	45.8	4.6	0.36
Carcasses graded Premium Choice	34.5	48.3	4.5	0.03
Carcasses graded Prime	1.70	0.841	1.335	0.65
Carcass with liver abscesses, %	7.82	7.76	2.50	0.98
Carcass classified as dark cutters, %	0.862	1.69	1.040	0.57

^1^Steers were purchased (day −1) from a commercial auction facility, transported for 280 km (4 h) and unloaded at the research yard. Steers were maintained as a single group for 24 h with access to fresh water and wheat hay. During initial processing on day 0, steers individually received 10-mL of the mBAS (Ferappease^®^; FERA Diagnostics and Biologicals; College Station, TX) or mineral oil (CON). Treatments were applied topically to the nuchal skin area (5 mL) and above the muzzle (5 mL) of each steer. Initial processing included administration of vaccines, anthelmintic, and growth-promoting implant (Synovex Choice^®^; Zoetis, Florham Park, NJ). On day 75, all steers were reimplanted (Synovex Plus^®^; Zoetis) and received another 10-mL dose of mBAS or CON according to treatment (5 mL in nuchal skin area and 5 mL above the muzzle).

^2^Trained personnel assessed carcass characteristics after a 24-h chill. Backfat thickness was measured at the 12^th^ rib; marbling score: 300 = Slight^00^, 400 = Small^00^; yield grade calculated according to USDA (1997). The dressing percentage was calculated by dividing hot carcass weight and final body weight (average day 138 and 139).

No treatment differences (*P *≥ 0.57) in proportion of carcasses with liver abscesses or classified as dark cutters were detected (Table 4). The incidence of liver abscesses in feedlot cattle is increased by consumption of highly fermentable finishing diets (Nagaraja and Lechtenberg. 2007). However, treatment effects on feed intake were insufficient to increase the incidence of this disorder in mBAS steers. Although tylosin was not supplemented (Nagaraja and Chengappa, 1998), the incidence of liver abscesses in this experiment (7.79%; 18 carcasses with liver abscess from 234 total carcasses) was less when compared with the average in US feedlot cattle (17.8%; Eastwood et al., 2017). Dark cutting carcasses are increased in cattle exposed to stressors near the time of slaughter, as cortisol depletes muscle glycogen and prevents the decrease of postmortem meat pH (Apple et al., 2005). The incidence of dark cutters herein (1.28%; 3 dark cutters from 234 carcasses) was also less when compared with values reported industry average (1.80%; Mayer et al., 2024), although this experiment was not designed to evaluate this response (Wulf et al., 2002; Mackey et al., 2024).

### Experiment 2

Initial BW and days on feed did not differ (*P *≥ 0.23) between treatments (Table 5), but these variables were significant covariates for all other analyses (*P *≤ 0.04). Bulls administered mBAS had greater (*P *≤ 0.02) ADG and final BW compared with CON bulls (Table 4). Mean feed intake was greater (*P *< 0.01) for mBAS vs. CON bulls, whereas G:F did not differ (*P *= 0.91) between treatments (Table 5). Similar to Exp. 1, the greater ADG and final BW of mBAS bulls were mostly driven by increased feed intake throughout the experimental period (treatment × day interaction; *P *= 0.55). As previously described, bulls were only handled for initial processing and when loaded for slaughter. For this reason, mBAS was administered at initial processing only because there were no handling events during the finishing phase expected to affect feed intake or growth performance. As in Exp. 1, carcass dressing did not differ (*P *= 0.87) between treatments and HCW was greater (*P *= 0.04) in mBAS bulls compared with CON (Table 5).

**Table 5.** Performance, carcass, and health parameters during the finishing period of yearling Nelore (*Bos indicus*) bulls (Exp. 2) administered the maternal bovine appeasing substance (**mBAS**; n = 10) or mineral oil as placebo (**CON**; n* *= 10) ^1^

Item	CON	mBAS	SEM	*P-value*
*Growth performance* ^2^				
Initial BW, kg	403.9	400.7	11.6	0.87
Days on feed, d	97.2	94.8	1.4	0.23
Final BW, kg	502.1	512.3	2.8	0.01
Average daily gain, kg/d	1.031	1.154	0.027	0.02
Feed intake, kg/d	9.15	10.0	0.35	<0.01
Gain to feed, kg/kg	0.113	0.116	0.006	0.91
*Carcass traits*				
Hot carcass weight, kg	281.4	287.5	2.7	0.04
Carcass dressing, %	56.1	56.1	0.3	0.87
*Health responses* ^4^				
Morbidity, %	15.0	7.11	2.20	0.02
Mortality, %	3.86	1.39	0.69	0.02

^1^Bulls were purchased (day −4) from a commercial ranch, transported for 1,800 km (96 h) and unloaded at a commercial feedyard on day 0. During initial processing (day 0), bulls individually received 10-mL of the mBAS (Ferappease^®^; FERA Diagnostics and Biologicals; College Station, TX) or mineral oil (CON). Treatments were applied topically to the nuchal skin area (5 mL) and above the muzzle (5 mL) of each bull.

^2^Body weight (**BW**) was recorded on day 0 (shrunk BW) and a 14% transport shrink was considered (Schwartzkopf-Genswein et al., 2016); therefore, initial BW was calculated as shrunk BW (day 0) × 1.14. Final BW (unshrunk) was recorded when loading bulls for slaughter. Initial BW and days on feed were used as independent covariates for all growth performance traits. Feed intake was evaluated by recording daily offer of the total mixed ration and measuring orts weekly from each pen, which was divided by the number of bulls within each pen and expressed as kg per bulls/d. Gain to feed was calculated using total BW gain (based on initial and final BW), and total feed intake (kg of dry matter) of each pen during the finishing period.

^4^Bulls were observed daily for health conditions including bovine respiratory disease (**BRD**) symptoms as Colombo et al. (2021), and treated for BRD with florfenicol antibiotic + flunixin meglumine (Resflor Gold, Merck Animal Health) at 1 mL/7.6 kg of BW.

Bulls used in this experiment were highly susceptible to stressors associated with the transition from grazing to feedlot systems because of their *B. indicus* genetics (Cooke et al., 2020; Mota-Rojas et al., 2024), particularly the 96-h road transport (Schwartzkopf-Genswein et al., 2016; Mendes et al., 2024). The magnitude of such stressors is directly associated with immunosuppression and increase risk of BRD (Duff and Galyean, 2007). Accordingly, morbidity and mortality rates observed herein were all associated with BRD, with values (Table 4) above industry average (6.13% morbidity and 0.21% mortality due to BRD in Brazil; Baptista et al., 2017). Data from Baptista et al. (2017) included cattle transported for up to 700 km, which explains the greater morbidity and mortality rates noted in this experiment. Both morbidity and mortality rates, however, were less (*P *= 0.02) in mBAS compared with CON bulls (Table 4). These outcomes corroborate previous research where mBAS administration alleviated the negative consequences of stress to immunity of high-risk cattle (Pickett et al., 2024), decreasing the incidence of BRD and mortality in this experiment by 52 and 64%, respectively. All bulls diagnosed with BRD only received 1 antimicrobial treatment, and either regained health or mortality occurred. Cattle diagnosed with BRD experience reduced feed intake and ADG, even when the first therapeutic antimicrobial treatment is successful (Blakebrough-Hall et al., 2020; Pickett et al., 2023). Therefore, decreased BRD incidence also contributed to the greater feed intake of mBAS bulls, resulting in improved ADG, final BW, and HCW compared with CON.

Fonseca et al. (2021) reported greater feed intake but not ADG and HCW in yearling Nelore bulls transported for 880 km, administered mBAS upon arrival, and placed on a finishing diet for 108 d. Cooke et al. (2020) also reported similar ADG during the first 45 d on feed in yearling Nelore bulls transported for 200 km that received mBAS or not at feedlot entry. Both studies did not report morbidity and mortality occurrence, nor increased physiological markers such as hair cortisol and serum haptoglobin concentrations upon feedlot entry. Perhaps the bulls evaluated in Cooke et al. (2020) and Fonseca et al. (2021) were not exposed to the same level of stress as the bulls in this experiment, explaining why mBAS administration did not improve growth performance in those studies.

### Overall Conclusions

Administering mBAS to finishing cattle placed on feed as yearlings improve their growth performance by increasing feed intake. Angus-influenced steers that received mBAS at initial processing and at the time of reimplant consumed more feed, particularly after reimplanting, resulting in greater ADG, HCW, and carcass quality. These steers were not considered high-risk given their age, BW at arrival, previous grazing history, and short transportation distance. In turn, mBAS administration during initial processing to Nelore (*B. indicus*) bulls transported for 96-h reduced morbidity and mortality, resulting in greater feed intake, ADG, and HCW. Accordingly, mBAS reduces the physiological consequences elicited by stressful management that impairs growth and immunocompetence of feedlot cattle (Cooke, 2017; Cappellozza and Cooke, 2022). Results from this study further support the use of mBAS to improve productivity of feedlot systems, including those that receive yearling cattle exposed to stressful management procedures.
